# Supplementary material for: Integrating structure-based machine learning and co-evolution to investigate specificity in plant sesquiterpene synthases
Source: PLoS Comput Biol. 2021 Mar 22;17(3):e1008197. doi: 10.1371/journal.pcbi.1008197 (PMC8016262; doi:10.1371/journal.pcbi.1008197)
Supplement: S1 Appendix — Results from different homology modelling runs. (PDF) [file pcbi.1008197.s001.pdf]

S1 Appendix

Homology Modelling

The structure-based predictor presented makes extensive use of homology models. 500 such models were made for each STS sequence, following which the three with the lowest N-DOPE score were retained for use by the predictor. The N-DOPE score or normalized DOPE score is an atomic distance-dependent statistical potential. It is widely used as a metric to identify correctly folded homology models, where models with an N-DOPE $<$ -1 are considered ”near-native”.

In order to ascertain the affects of different templates and different structural regions on the modelling process we created models using three different approaches

1. Modelling the C-terminal domain region using the closest template, based on sequence identity, from the six structures in Table 2.

2. Modelling the C-terminal domain region using all six template structures.

3. Modelling the entire protein using all six template structures.

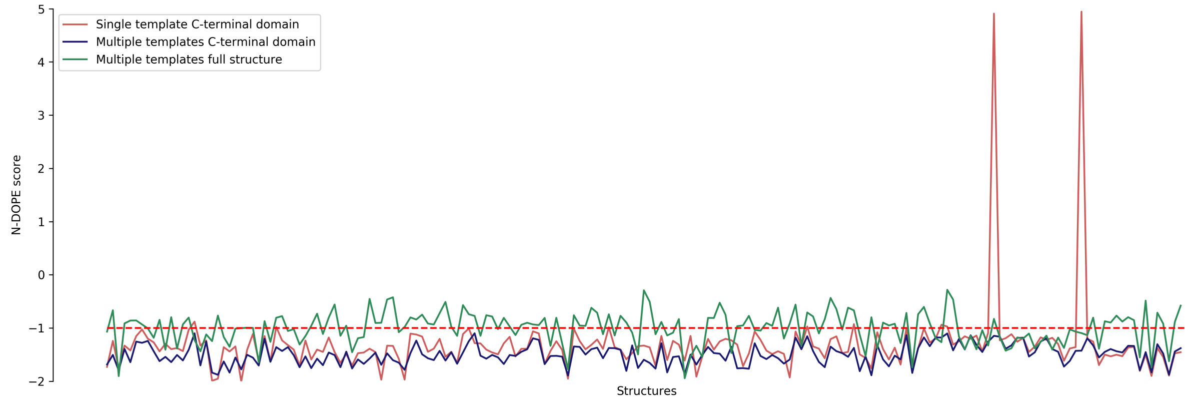

Figure 1: Comparison of N-DOPE scores across three different modelling approaches. The threshold below which models are considered near-native is depicted in red.

S1 Appendix Fig 1 compares the best (lowest) N-DOPE scores of these three approaches for all the modelled structures. The failure of approach 3 can be explained by the lack of a good sequence alignment in regions around the C-terminal domain due to highly varying sequences. It is clear that approach 2, modelling the C-terminal domain with multi-template modelling, performs best overall and also allows for more effective comparison across models. Therefore these are the models used for prediction.

While the N-DOPE score is a good measure of stable structures with plausible folding, it may not give the full picture in terms of similarity to the true structure. To gain more insight on this we also created multi-template models of the templates themselves, using the remaining five structures as templates for each. Comparing these template models to the real structures revealed that in four out of the six cases the models were less than 1 Å different from the real structures, while the remaining two were 2.5 Å different. This suggests that multi-template modelling using the six available STS structures truly captures structural characteristics of the STS enzymes being modelled.
