# Supplementary material for: Integrating structure-based machine learning and co-evolution to investigate specificity in plant sesquiterpene synthases
Source: PLoS Comput Biol. 2021 Mar 22;17(3):e1008197. doi: 10.1371/journal.pcbi.1008197 (PMC8016262; doi:10.1371/journal.pcbi.1008197)
Supplement: S2 Appendix — Fragmentation patterns of identified peaks from chromatograms and of corresponding reference compounds for Citrus bergamia STSs. (PDF) [file pcbi.1008197.s002.pdf]

S2 Appendix

Fragmentation Patterns

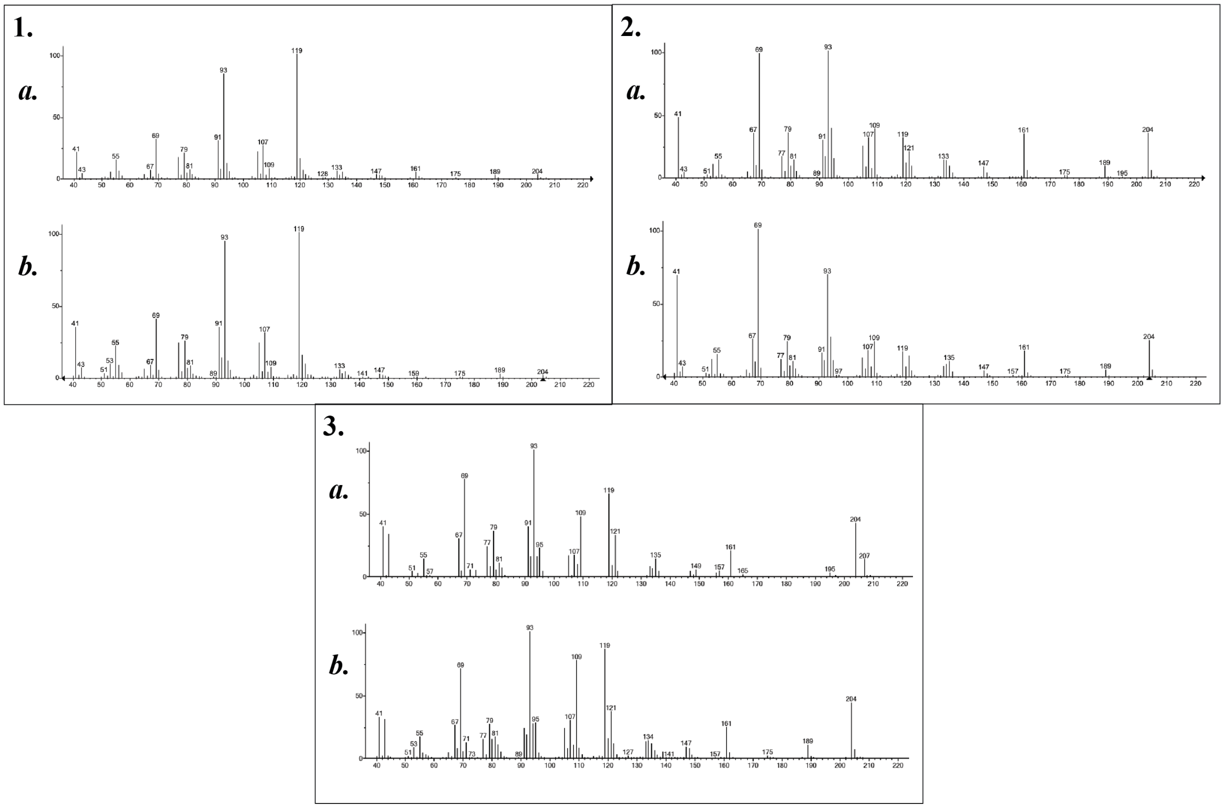

Figure 1: **Fragmentation patterns of the peaks identified in the chromatograms and of the corresponding reference compounds for MT636927 1a.** fragmentation pattern of peak 1; **1b.** fragmentation pattern of *trans*-α-bergamotene; **2a.** fragmentation pattern of peak 2; **2b.** fragmentation pattern of β-bisabolene; **3a.** fragmentation pattern of peak 3; **3b.** fragmentation pattern of α-bisabolol; **4a.** fragmentation pattern of peak 4; **4b.** fragmentation pattern of sesquisabinene; **5a.** fragmentation pattern of peak 5; **5b.** fragmentation pattern of zingiberene.

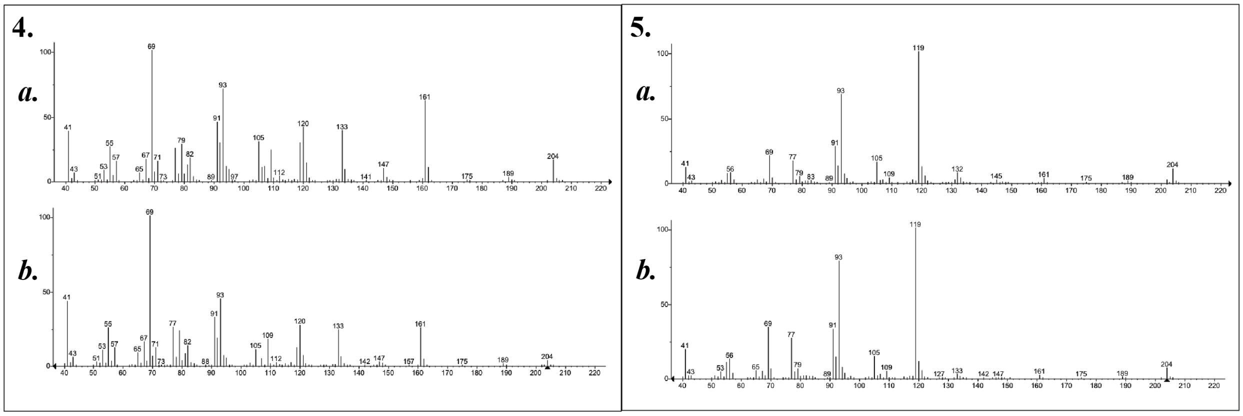

Figure 2: **Fragmentation patterns of the peaks identified in the chromatograms and of the corresponding reference compounds for MT636928 4a.** fragmentation pattern of peak 4; **4b.** fragmentation pattern of sesquisabinene; **5a.** fragmentation pattern of peak 5; **5b.** fragmentation pattern of zingiberene.

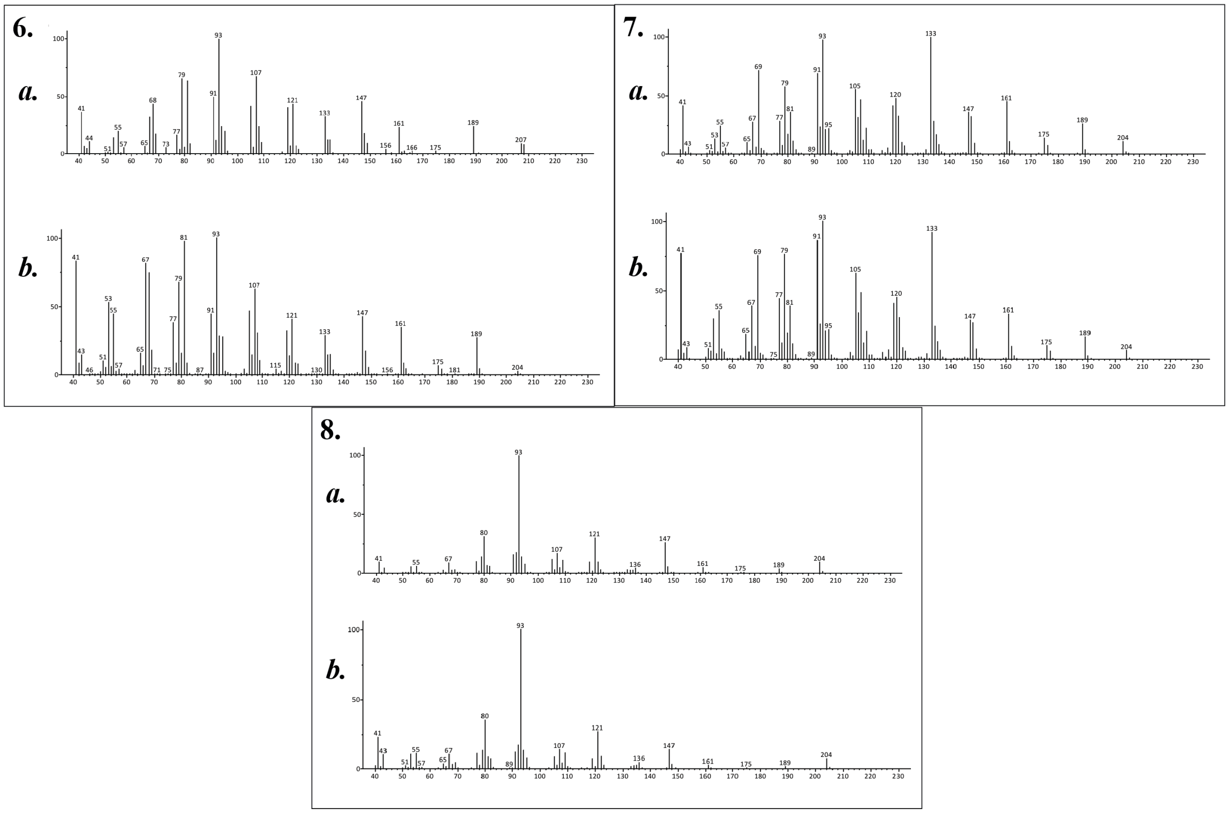

Figure 3: **Fragmentation patterns of the peaks identified in the chromatograms and of the corresponding reference compounds for MW384854 6a.** fragmentation pattern of peak 6; **6b.** fragmentation pattern of β-elemene; **7a.** fragmentation pattern of peak 7; **7b.** fragmentation pattern of β-caryophyllene; **8a.** fragmentation pattern of peak 8; **8b.** fragmentation pattern of α-caryophyllene;
