# Supplementary material for: Integrating structure-based machine learning and co-evolution to investigate specificity in plant sesquiterpene synthases
Source: PLoS Comput Biol. 2021 Mar 22;17(3):e1008197. doi: 10.1371/journal.pcbi.1008197 (PMC8016262; doi:10.1371/journal.pcbi.1008197)
Supplement: S1 Fig — Nerolidyl prediction percentages returned by Clf-str on characterized STSs calculated using the genus-based split. (PDF) [file pcbi.1008197.s004.pdf]

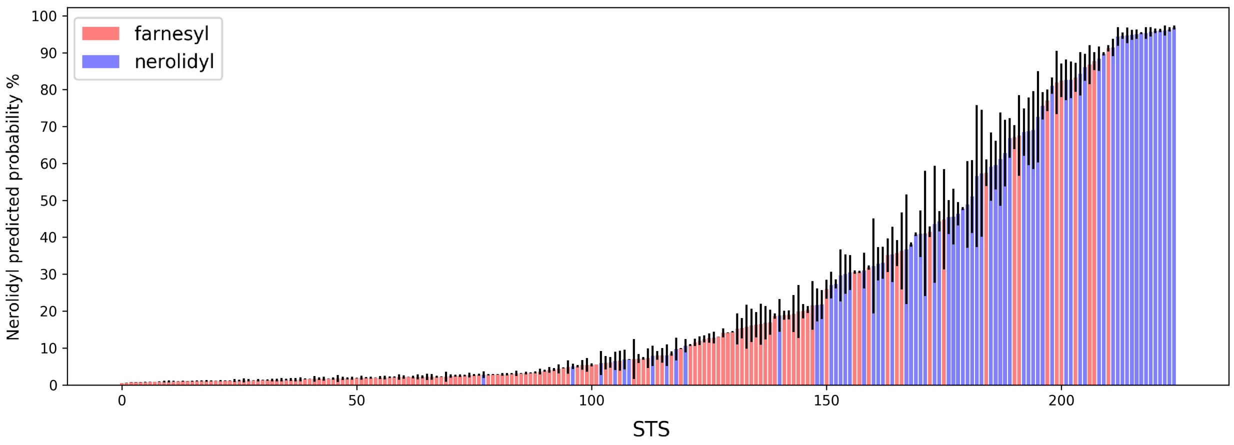

Figure S1: **Predicted nerolidyl percentages** Nerolidyl prediction percentages returned by Clf-str on characterized STSs calculated using the genus-based split.
