# Supplementary material for: Integrating structure-based machine learning and co-evolution to investigate specificity in plant sesquiterpene synthases
Source: PLoS Comput Biol. 2021 Mar 22;17(3):e1008197. doi: 10.1371/journal.pcbi.1008197 (PMC8016262; doi:10.1371/journal.pcbi.1008197)
Supplement: S2 Fig — Residue scores found by Clf-str across 10 different train-test splits based on genus. (PDF) [file pcbi.1008197.s005.pdf]

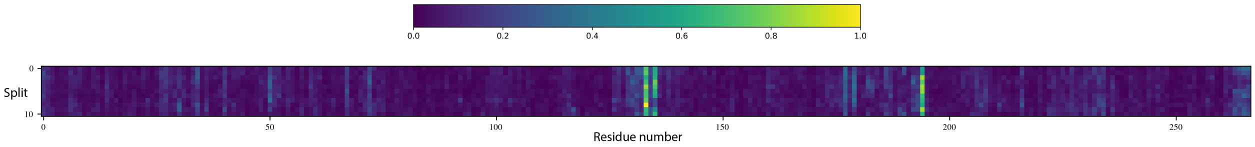

Figure S2: **Residue importance scores** Residue scores found by Clf-str across 10 different train-test splits based on genus.
