# Supplementary material for: Integrating structure-based machine learning and co-evolution to investigate specificity in plant sesquiterpene synthases
Source: PLoS Comput Biol. 2021 Mar 22;17(3):e1008197. doi: 10.1371/journal.pcbi.1008197 (PMC8016262; doi:10.1371/journal.pcbi.1008197)
Supplement: S3 Fig — A. Scores of predicted contacts from co-evolutionary analysis in decreasing order. The 1500 contacts on the left of the orange dashed line are considered in the text. b. Pairwise minimum β-carbon distance matrix (in Å) across all six template structures in Table 1 for the residue positions present in the tobacco aristolochene synthase (TEAS) structure. c. The top 1500 predicted co-evolving contacts on the TEAS structure, indicated in black. (PDF) [file pcbi.1008197.s006.pdf]

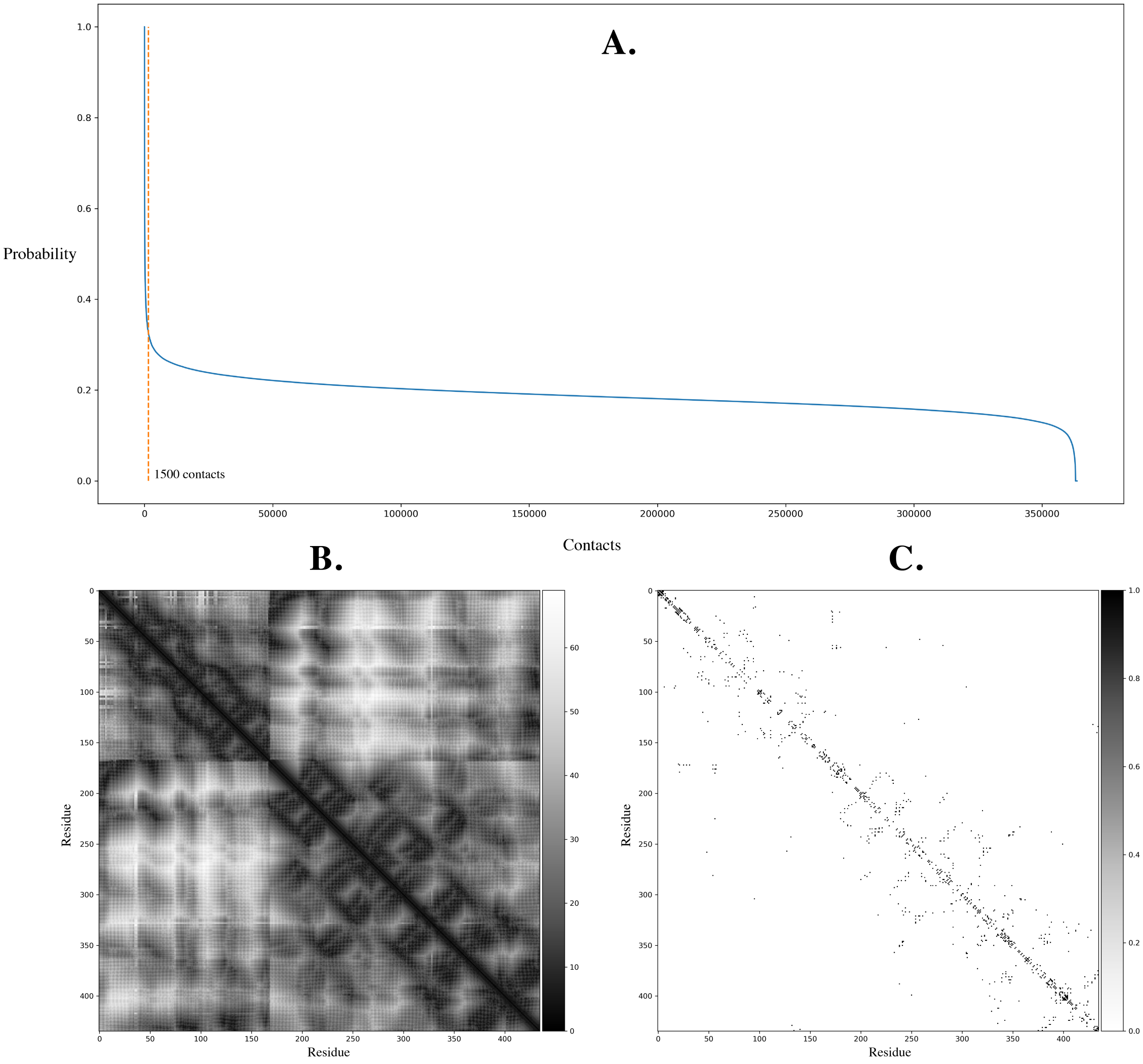

Figure S3: **Predicted contacts from co-evolutionary analysis A.** Scores of predicted contacts from co-evolutionary analysis in decreasing order. The 1500 contacts on the left of the orange dashed line are considered in the text. **B.** Pairwise minimum β-carbon distance matrix (in Å) across all six template structures in Table 2 for the residue positions present in the tobacco aristolochene synthase (TEAS) structure. **C.** The top 1500 predicted co-evolving contacts on the TEAS structure, indicated in black.
