# Supplementary material for: Integrating structure-based machine learning and co-evolution to investigate specificity in plant sesquiterpene synthases
Source: PLoS Comput Biol. 2021 Mar 22;17(3):e1008197. doi: 10.1371/journal.pcbi.1008197 (PMC8016262; doi:10.1371/journal.pcbi.1008197)
Supplement: S4 Fig — Sequence alignment of tobacco aristolochene synthase with STS examples discussed in text. (PDF) [file pcbi.1008197.s007.pdf]

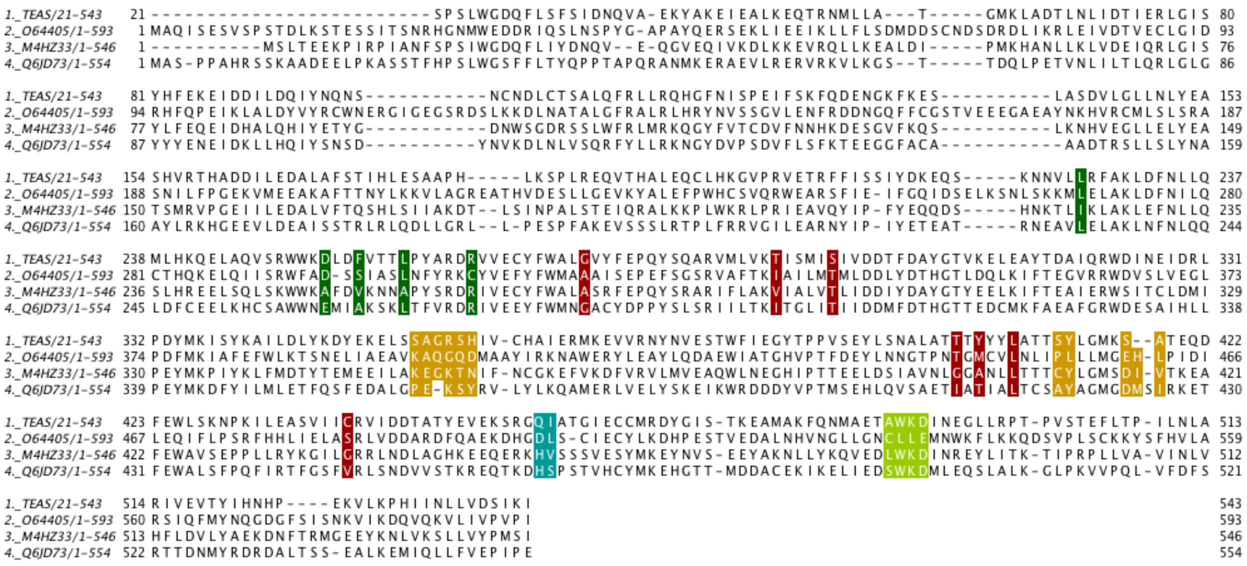


Figure S4: **Alignment of discussed STSs** Sequence alignment of tobacco aristolochene synthase with STS examples discussed in text.
