# Supplementary material for: Integrating structure-based machine learning and co-evolution to investigate specificity in plant sesquiterpene synthases
Source: PLoS Comput Biol. 2021 Mar 22;17(3):e1008197. doi: 10.1371/journal.pcbi.1008197 (PMC8016262; doi:10.1371/journal.pcbi.1008197)
Supplement: S5 Fig — Chromatograms obtained from the R. sphaeroides strain expressing a. MT636927, b. MT636928, and c. MW384854 with peaks labelled. (PDF) [file pcbi.1008197.s008.pdf]

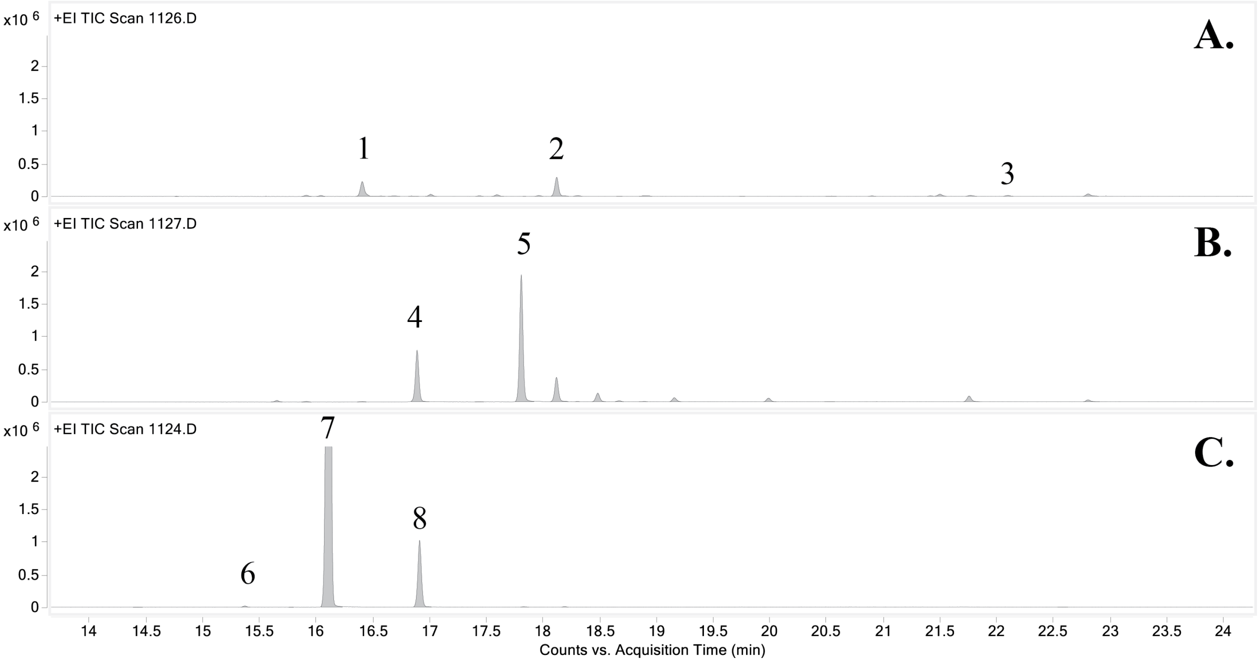

Figure S5: ***Citrus bergamia* STS chromatograms** Chromatograms obtained from the *R. sphaeroides* strain expressing **A.** MT636927, **B.** MT636928, and **C.** MW384854 with peaks labelled.
