# Supplementary material for: Integrating structure-based machine learning and co-evolution to investigate specificity in plant sesquiterpene synthases
Source: PLoS Comput Biol. 2021 Mar 22;17(3):e1008197. doi: 10.1371/journal.pcbi.1008197 (PMC8016262; doi:10.1371/journal.pcbi.1008197)
Supplement: S6 Fig — Classification framework of gradient boosting trees, to deal with a large number of residue-based features as well as allow for intuitive selection of the most predictive residues. A separate gradient boosting tree (GbT-s) is trained on each kind of feature across all aligned residue indices. The weights obtained from all the GbT-s classifiers are pooled (by taking the sum of the normalized weights) to select the top 30 residues. All feature values of these selected residues are used as input to the final classifier GbT. The parameter settings of the GbT-s and GbT classifiers are listed in Materials and Methods. (PDF) [file pcbi.1008197.s009.pdf]

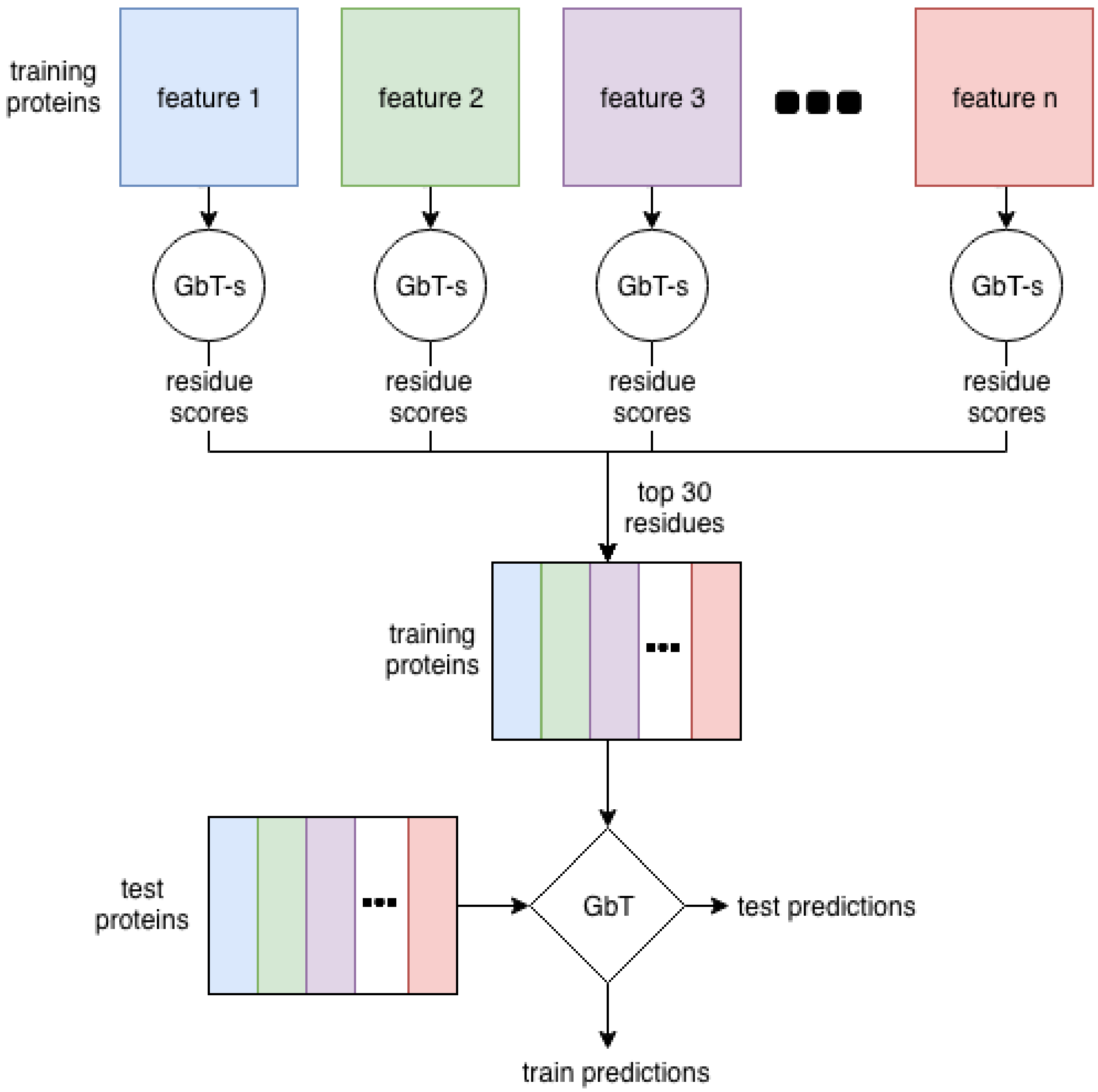

Figure S6: **Classification framework** Classification framework of gradient boosting trees, to deal with a large number of residue-based features as well as allow for intuitive selection of the most predictive residues. A separate gradient boosting tree (GbT-s) is trained on each kind of feature across all aligned residue indices. The weights obtained from all the GbT-s classifiers are pooled (by taking the sum of the normalized weights) to select the top 30 residues. All feature values of these selected residues are used as input to the final classifier GbT. The parameter settings of the GbT-s and GbT classifiers are listed in Methods.
