# Supplementary material for: Integrating structure-based machine learning and co-evolution to investigate specificity in plant sesquiterpene synthases
Source: PLoS Comput Biol. 2021 Mar 22;17(3):e1008197. doi: 10.1371/journal.pcbi.1008197 (PMC8016262; doi:10.1371/journal.pcbi.1008197)
Supplement: Attachment — Submitted filename: Reply_to_Reviewers.pdf [file pcbi.1008197.s010.pdf]

Dear Editor,

Two reviewers commented on our manuscript; we thank both of them for their comments and suggestions. We believe the revisions made in response to these suggestions have greatly improved the impact and clarity of our results. Please find below a point-by-point response to the comments. Our response is shown in italic. Textual changes are marked in red in the manuscript.

Reviewer #1: This manuscript describes a computational approach to predict the carbocationic intermediate specificity of plant sesquiterpene synthases. While several previous studies have investigated residues responsible for product specificity by using mutant based approaches, the novelty of this work is a combined approach of homology modeling, machine learning, and co-evolutionary analysis. The authors take advantage of a large number of already characterized sesquiterpene synthases to define residues that contribute to cation intermediate specificity. While the approach does not predict specific sesquiterpene product outcomes it appears useful for facilitating screenings of terpene synthases with products derived predominantly from a farnesyl or nerolidyl cation intermediate. This type of analysis could be useful in similar predictions for other C10 or C20- terpene synthases. Overall, the manuscript is well written and presented and I would like to add only minor comments and concerns.

We agree with these remarks and in particular with the assessment that our approach is useful for facilitating screening of terpene synthases.

1. The specificity predictions for uncharacterized sesquiterpene synthases from Citrus were between 10 and 50%. It would be advantageous to compare these results in terms of their robustness with predictions for enzymes that make products predominantly from farnesyl cations.

We thank both reviewers for this suggestion as this result indeed makes the interpretation of screening results clearer. We have now added Supplementary Figure S2 showing the predicted nerolidyl percentage across all characterized enzymes using the genus-based validation scheme. The figure shows that nerolidyl cation-specific enzymes do indeed have generally lower predicted percentage, in line with the values observed for the Citrus enzymes, which is not surprising given the lower number of nerolidyl cation-specific STSs in the training dataset (emphasized in lines 190-197). We now also refer to this figure when talking about the 10% threshold used in the Citrus screening application (lines 371-373). We have also added a few lines on the various metrics used (lines 180-182 and lines 514-527).

2. Salmon et al. (2015) described a residue network required for a transition from a linear to cyclic sesquiterpene product. Although this study followed a different goal, I am wondering whether the authors found and overlap in the residues that were investigated.

We thank the reviewer for this suggestion. We have investigated this and found 2 out of 3 residues mentioned by Salmon et al. in our top predictive residue set. We have now added

this study to the others (lines 292-294) and added some lines emphasizing how our integrative data-driven approach finds a combination of residues found by individual smaller scale mutagenesis approaches. (lines 315-319)

3. Fig. 1 It should be (E,E)-alpha-farnesene. FPP should be labeled (E,E)-FPP.

We changed this. (line 87, Fig 1 caption)

4. What do the numbers in white boxes represent in Fig. 4c? Carbon atoms?

Yes, these are carbon atoms according to the numbering of the FFF substrate analog moeity in the tobacco aristolochene synthase PDB, we have added this to the figure caption. (Fig 4 caption)

5. Table S1 lists four beta-caryophyllene synthases from Acanthopanax sieboldianus. I am surprised that the product outcome of four enzymes is so similar; or do these enzymes make mixtures of products with beta-caryophyllene as major compounds?

This was an error on our side as these 4 enzymes are isoforms with 99% identity and only one was characterized in the study, we have removed the others from the set and added 21 more newly characterized enzymes from three different genera. Thus, the final test set now has 42 enzymes which we believe is a more representative set that further emphasizes the good performance of our predictor. (lines 215-218, line 528, lines 624-625, S1 Table)

6. The authors use the terms farnesyl and nerolidyl synthases. Since "farnesyl" and "nerolidyl" are not end products of these enzymes, I would better use the terms farnesyl cation- or nerolidyl cation-specific synthases.

Changed all throughout the text

7. Line 237: ...show the predicted contact...

Changed (line 259)

8. Line 350: "STSs with desired product specificity" is not absolutely correct since the analysis predicts intermediate specificity but not the final product profile.

Changed to desired intermediate specificity (line 388)

Reviewer #2: This manuscript describes an interesting but clearly underdeveloped approach to computational analysis for functional assignment in the large sub-family of

plant sesquiterpene synthases (STSs). The STSs represent an intriguing target for such analysis, with thousands of putative members, but only a couple of hundred that have been functionally (biochemically) characterized. Those characterized were previously assembled into a database by the authors, leading to the finding that the multiple products often observed in this sub-family still largely result from a single initial conformation of their common substrate, farnesyl diphosphate (FPP). Thus, even these promiscuous STSs generally exhibit some specificity in substrate folding/conformation within their active sites. Here the authors build on this finding. In particular, this pre-catalytic conformation directs the ensuing carbocation into certain reaction 'channels', first splitting between immediate cyclization (or terminating deprotonation) of the initially formed farnesyl carbocation versus isomerization to the nerolidyl carbocation that has additional cyclization options, with each of the subsequent cyclization options enumerated to allow assignment of reaction pathways (e.g., farnesyl is F1, nerolidyl is N1, with 10,1-cyclization of F1 then assigned as F11, while that of N1 as N11). This was coupled with protein modeling to identify thirty residues that correlate with pathway/carbocation. However, it is unclear why thirty were chosen. Examination of the sequence logos for these thirty for the first level of prediction (F1 vs N1), indicates that many of these positions do not seem significantly different (226, 253 & 260), while others do (e.g., 293 and 298).

We realize that the sequence logos (Fig.3) might give a somewhat wrong impression. One of the main findings of our experiments is that structural information improves prediction performance. The sequence logos contain residues which were found as informative by the structure-based predictor. Some of these residues have clear differences in their sequence composition, but others are found predictive despite not having differences in their sequence composition. This indicates that their differences lie in their structural features or because of their structural interaction with neighbouring residues. We have added some lines to stress this point in the text (lines 232-238 and lines 59-60) and also made this clearer in the figure caption (Fig 3 caption).

The authors do discuss previously reported mutational analyses that seem to support the relevance of some of these residues, but these are obviously selected examples. It would be much more convincing if the results were used in predictive fashion and then tested.

We attempted a search through literature to find mutational studies that changed the initial cation specificity of a plant STS to demonstrate that the residues found by our data-driven integrative approach contain many of the residues found by these individual small-scale studies (lines 315-317) as well as pinpoint those which generalize across all species. We acknowledge the reviewer's concern that there is a risk for bias, but we did our best to avoid any such bias in our final list. As far as we know we have not wilfully excluded any relevant study (note that while there are many mutational studies on plant STSs in general, we are in particular focusing only on studies where the initial parent cation of the product is changed from farnesyl to nerolidyl or vice versa). Independently, two of the authors performed searches for more such publications and found two additional relevant mutational studies. One involves a residue which is not found by our predictor (added to text, lines 308-310); the other, also suggested by reviewer #1, involves three residues of which two are found by our predictor (lines 292-294). Both are now added to the manuscript. In addition, we added the result of a hypothesis test (Fisher exact test) for the

significance of the number of residues found both by our predictor and in literature (p ~ 9.8e^{-07}) (lines 317-319).

Also, while we agree that these mutational studies support the relevance of some residues, especially those very close to the active site, many of the residues we found have not yet been explored by such studies and we believe that their importance is proven by the good predictive performance of our approach on the different validation sets and on new enzymes such as those in the independent test set (lines 215-224).

For example, such analysis suggests that most of the cadalane producing STSs actually proceed via farnesyl rather than nerolidyl, although this would then require several more steps/intermediates. Could this be tested by labeling (e.g., in collaboration with Prof. Jeroen Dickschat)?

We would indeed be happy to start such collaboration with the group of Professor Dickschat, but we do not feel this should be part of the current manuscript. STSs producing only cadalane products are only a small number of cases, too few to be informative to the predictor or have an impact on screening applications. Hence, they are only a minor issue in manuscript and are excluded from the training set.

Since STSs marked as cadalane could either be an artefact of experimental conditions, derive from the farnesyl cation, or derive from the nerolidyl cation, labelling experiments such as the one proposed would require a significant amount of experimentation, while some unclarities may still persist. Thus, we do not believe that the proposed experiment is within the scope of our analysis.

The MDS plot in Figure 2 shows where these ambiguous cases lie according to the features of their predictive residues, but its interpretation is not clear-cut (added to the text, lines 209-210) as the dots on the farnesyl side of the plot could represent farnesyl cation-derived cadalane synthases or farnesyl cation-derived germacrene D synthases.

Part of the concern is that the chosen arena for prediction, predictive analysis of STSs from Citrus bergamia, provided only modest (at best) results.

We recognize the concern of the reviewer about the value of the Citrus bergamia application. However, the Citrus bergamia screening application is not the only chosen area for prediction. We have also demonstrated prediction results on a test set of novel enzymes from literature which were not used in training the model. We have now supplemented this test set with additional enzymes to a total of 42 enzymes from a variety of different species many of which are not present in the training set (lines 215-224, S1 Table). We believe that these results further demonstrate the superior predictive performance of our predictor.

Even considering the Citrus bergamia case, predicting specificity in STSs is a very difficult task due to their diversity. We believe that narrowing down the number of enzymes needed to be characterized from 11 to 3 (out of which 2 are correct) is a significant improvement upon the oft-used sequence identity screening which predicts all 11 as farnesyl cation-derived synthases. This also serves as a demonstration of how researchers in the field can use our approach to their own species of interest.

In particular, of the three STSs predicted to proceed via N1 that were amenable to experimental characterization, apparently only two actually did so. Moreover, one of these two only had 11% predicted nerolidyl specificity! This seems extremely low, and begs the question of what the predicted specificity was for the STS that apparently did not proceed via N1 (this data is not presented but needs to be, both prediction and actual products).

It would also be helpful to present the distribution of all STSs (and just those from C. bergamia) for predicted nerolidyl specificity, to better judge how well this approach does in distinguishing between those that carry out even this initial carbocationic transformation.

We agree that without the data of distribution of predicted probabilities, these percentages were difficult to interpret. We have now added Supplementary Figure S2 which shows the predicted nerolidyl percentages for all characterized enzymes. The lower percentages of nerolidyl cation-specific enzymes are expected due to the lower numbers of these enzymes in our training dataset. We have now explained this more clearly in the text (lines 190-197) and referred to this additional figure when discussing the Citrus results (lines 371-373). We have also added the data for the third predicted Citrus bergamia enzyme that does not produce nerolidyl cation-derived products (line 377, lines 381-382, S6 Fig – also changed to have the same Y-axis range across all three genes, S7 Appendix).

In any case, the results are well short of having the sort of resolution that would be desired for any sort of accuracy in predicting STS catalytic activity (i.e, product outcome), which would require drilling down several additional steps in the catalyzed reaction. On the other hand, if more evidence can be provided supporting the claimed accuracy for predictive separation of STSs proceeding via N1, this would nevertheless represent a reasonable advance in this area.

While predicting product specificity is a difficult goal, we believe that this work provides the first step towards obtaining such higher resolution predictions along the catalytic reaction. Even with this first step, we were able to look only at bisabolyl cation specific STSs in Citrus bergamia by excluding the two enzymes which clustered close to the nerolidol cluster (line 374), starting with a set of 11 predicted STSs of which three were selected and experimentally verified. This is in strong contrast with the result of sequence identity-based screening which would predict all these 11 enzymes as farnesyl cation-specific. Our results on the test set of 42 enzymes from literature as well as the separation in Figure 2 and the predicted percentages in Supplementary Figure S2 also give strong evidence of the accuracy of separation of STSs based on their parent cation. Thus, we believe that this paper aimed at describing a computational approach for predicting STSs proceeding via N1 provides the advance in the area that the reviewer speaks of.

Have all data underlying the figures and results presented in the manuscript been provided?

Large-scale datasets should be made available via a public repository as described in the *PLOS Computational Biology* data availability policy, and numerical data that

underlies graphs or summary statistics should be provided in spreadsheet form as supporting information.

Reviewer #1: Yes

Reviewer #2: **No:** See comments regarding presentation of predicted specificity.

We now added the requested information on the predicted specificity.
